# Reassessing socioeconomic inequalities in mortality via distributional similarities

**DOI:** 10.1186/s12963-025-00365-1

**Published:** 2025-02-22

**Authors:** Ana C. Gómez-Ugarte, Ugofilippo Basellini, Carlo G. Camarda, Fanny Janssen, Emilio Zagheni

**Affiliations:** 1 Max Planck Institute for Demographic Research, Rostock, Germany; 2 Institut national d’études démographiques, Aubervilliers, France; 3 Netherlands Interdisciplinary Demographic Institute (NIDI) - KNAW, University of Groningen, The Hague, The Netherlands; 4 Population Research Centre, Faculty of Spatial Sciences, University of Groningen, Groningen, The Netherlands

## Abstract

Commonly used measures of socioeconomic inequalities in mortality, such as the slope and the relative index of inequality, are based on summary measures of the group-specific age-at-death distributions (e.g. standardized mortality rate or life expectancy). While this approach is informative, it ignores valuable information contained in the group-specific distributions. A recent approach applied a measure of distributional dissimilarity (the non-overlap index) to measure lifespan stratification. In this paper, we rigorously evaluate and further implement the multi-group extension of the non-overlap index ($S^P$) to measure socioeconomic inequalities in mortality across a number of groups, and assess whether differences across countries and over time are driven by mortality or compositional changes in two applications with different data availability: educational groups (Sweden and Denmark) and groups defined by an area-level deprivation measure (England). Our findings suggest that the multi-group $S^P$ is sensitive not only to changes in the means or variances, but also to broader mortality changes that affect distributional shapes. The method can be employed to any context where mortality rates by age are available by sub-groups. Furthermore, levels and trends in mortality inequalities computed with the multigroup $S^P$ often differ compared to other conventional summary-based measures. Moreover, we find that the contribution of mortality changes to changes in inequalities is generally greater than that of the changes in the population composition. Whereas levels and trends of inequalities may depend on whether life expectancy- or lifespan variation-based measures are employed, the multi-group $S^P$ provides a holistic perspective by capturing both dimensions simultaneously.

**Supplementary Information:**

The online version contains supplementary material available at 10.1186/s12963-025-00365-1.

## Introduction

A long-standing literature has shown that patterns of mortality can drastically differ between groups in a population and across geographical areas inside a country, producing socioeconomic inequalities in mortality [13, 16, 19]. Evidence suggests that socioeconomic disadvantage often results in health disadvantage, leading to a social gradient in mortality. Low educated groups or those living in deprived areas tend to have lower life expectancy–the average number of years a person is expected to live if the current mortality conditions prevail through their entire life–, and higher lifespan variation–the variability in the age-at-death among individuals in a population—[17, 22, 36, 43]. This finding has sparked interest in measuring differences in mortality between groups within a population or country.

Different measures to evaluate socioeconomic inequalities in health and mortality have been proposed in the literature. These measures vary in complexity, in their incorporation of different population groups, and more importantly, in conceptual issues and implicit assumptions about inequality. Several studies have analyzed the strengths and limitations of different measures of health or mortality inequality [11, 18, 30, 31].

Table 1 presents the most commonly used approaches to measure socioeconomic inequality in mortality in the field of demography and population health, along with their descriptions and properties. Perhaps the simplest methods are the range and ratio measures, whereby inequality is computed by looking only at the most and least advantaged groups, disregarding any information from other groups. To overcome this issue, measures that account for all subgroups, such as the slope and relative index of inequality (SII and RII), were developed [18, 26, 29]. These measures quantify the social gradient in mortality by a weighted regression between the subgroups’ mortality measures (generally the age-standardized mortality rate) and their relative rank in terms of socioeconomic status. Other important measures used in the broader field of socio-economic inequalities in mortality are the average inter-group difference (AID) [39], the population attributable risk (PAR) and the population attributable fraction (PAF) [11].

**Table 1:** Common measures of socioeconomic inequalities in mortality

Measure	Formula and description	Properties
Range/Ratio	$\text {Range} = m_k - m_1$ $\text {Ratio} = \frac{m_k}{m_1}$ The difference/ratio of the measure of mortality between the most advantaged and the least advantaged group.	It is a simple and readily interpretable measure, and it can be applied to non-ordinal socioeconomic variables. However, it only reflects information of the extreme groups.
Slope index of inequality	$m_i=\alpha + \beta R_i$ $\text {SII} = \hat{\beta }$ It is the slope coefficient ($\hat{\beta }$) of the regression line between the group-specific mortality measure against their relative rank of socioeconomic status.	It measures the socioeconomic gradient in the mortality measure. It reflects the patterns of all social groups and considers the proportion of population in each group. It is often estimated by weighted least square regression, though other models have been proposed. However, it can only be applied to ordered groups.
Relative index of inequality	$m_i=\alpha + \beta R_i$ $\text {RII} = \frac{\hat{\beta }+\hat{\alpha }}{\hat{\alpha }}$ It is the relative counterpart of the SII. It can also be estimated as $\hat{\beta }/\bar{m}$.	

Let $m_i$ be the mortality measure (life expectancy, lifespan disparity, median age at death, etc.), $w_i$ the population share and $R_i = \frac{1}{2}w_i + {\sum }_{j=1}^{i-1}w_j$ the relative rank of group *i*. Where $i \in 1,..,k$, and *k* is the number of groups, and $\bar{m}$ the mean of the mortality measure of all groups. [11, 26, 29]

When applied to mortality, these commonly used measures of socioeconomic inequality are based on summary measures of mortality for each group, such as age-standardized mortality rates, life expectancy, modal age at death and, more recently, lifespan variation measures [11]. Previous studies have applied them to estimate the range of life expectancy for the high and the low-educated groups [48], range and ratio of the age-standardized mortality rates [22], the slope and the relative indexes of inequality of lifespan variation [37], or the difference between mortality indicators (life expectancy and lifespan variation) of each group and those of the overall population [44], among others.

These summary measures are convenient as they are easily interpretable and because they summarize each group’s mortality information into a single number. However, this approach can hide important differences in the underlying mortality patterns, and therefore provide an incomplete or biased assessment of socioeconomic inequalities in mortality. For example, one recent study showed that comparing distributional differences in mortality of groups defined by income quintiles in Finland revealed different patterns than those derived from the comparison of life expectancy and lifespan variation [38].

**Fig. 1:**
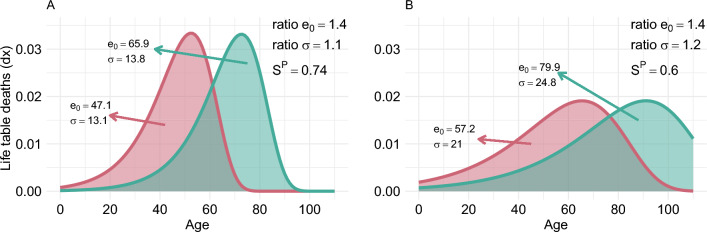
Hypothetical scenarios of the age-at-death distributions of two equal-sized (Panels A and B) population’s subgroups. *Note:* The annotations on the graphs include: life expectancy at birth ($e_0$), lifespan variation (measured by the standard deviation of the ages-at-death, $\sigma$), ratio in life expectancy at birth between both distributions (ratio $e_0$), ratio in standard deviation of the ages-at-death between both distributions (ratio $\sigma$) and pairwise non-overlap index ($S^P$). *Source:* Authors’ own elaborations

To illustrate this, we present two hypothetical populations in Fig. 1 along with their respective level of socioeconomic inequality in mortality derived from different measures. The figure shows two sub-populations (of equal size) at two points in time or from two different countries. The ratio of life expectancy between the subgroups is the same in both panels and equal to 1.4, suggesting constant differences. Furthermore, the ratio in lifespan variation, measured with the standard deviation of the ages-at-death, increases from 1.1 to 1.2 between panels A and B. However, the overlap of the two distributions is greater in panel B than in panel A, indicating that more people share similar lifespans, or equivalently, that there is more equality between the two subgroups. These conclusions also hold in the analysis of absolute measures of inequality (see Fig. S1 in the Supplementary Materials).

This example suggests that patterns of convergence/divergence between groups may be hidden when the measurement of socioeconomic inequality in mortality is restricted to the comparison of summary measures of mortality. Furthermore, given the regular shape of human mortality, there is a large overlap between different socioeconomic groups’ age-at-death distributions, which have more similarities than dissimilarities. In other words, many individuals from different groups can have similar lifespans [46]. Consequently, relying solely on age-standardized mortality rates, life expectancy, lifespan variation, or a combination of them may be insufficient to evaluate socioeconomic inequalities in mortality.

One recent study by [38] proposed the use of a statistical distance metric, the non-overlap index, to measure lifespan stratification –the extent to which social groups form unique and distinguishable strata across age-at-death distributions–between groups. The authors present two formulas on how their method could be extended to multiple populations, but barely employ them in their analysis which instead focuses on the comparison between the lowest and highest groups. Here, we rigorously evaluate the properties, and empirically apply the multi-population extensions proposed by [38] to measure between-group socioeconomic inequality in mortality. We apply the measure to two contexts with different data availability: educational groups (Sweden and Denmark) and groups defined by an area-level deprivation measure (England). Additionally, using decomposition analysis, we analyse whether changes over time in inequalities are driven by mortality or by compositional changes.

## How has the age-at-death distribution been used before?

Over time demographers have recognized the value of the information contained in age-at-death distributions and have used it to answer different research questions related to mortality inequalities. Is mortality converging across countries or across socioeconomic groups [8, 35]? What is the probability that an individual in one population outlives an individual in another population [46]? What is the degree of stratification of lifespans by social characteristics [38]? We recover the arguments put forward by previous studies to answer the question: Can the measurement of socioeconomic inequalities in mortality be refined by using the whole information of the age-at-death distribution?

Statistical distance or divergence measures can be employed to estimate the distance or similarity between two distributions, or in our case, two age-at-death distributions. Some previously used measures in demography are the Shannon entropy [3], the Tanimoto or Jaccard index [38] and Kullback–Leibler divergence (KLD), the latter being the most frequently used [5, 8, 35].

The KLD or relative entropy is a measure of distributional divergence frequently used in the field of information theory. It quantifies the amount of information that would be lost if one distribution is used to estimate another. In demography, it has been used to evaluate mortality convergence across countries [5, 8] and between education groups [35]. Under near-normality assumptions, the KLD can be decomposed into two parts: one reflecting differences in means and one reflecting differences in variances [34]. Evidence from this decomposition varies according to the groups analysed. When looking at mortality convergence across countries, groups vary mostly because of differences in standard deviation of the ages at death [8]. Conversely, education groups vary mainly due to differences in means [35]. The KLD has the limitation that it relies on the subjective choice of a reference distribution. Moreover, it is asymmetric, meaning that the KLD from distribution A to B is typically different from the KLD from B to A. Consequently researchers need to define a reference population, with common choices being the period average distribution or the population with the highest life expectancy or the lowest lifespan variation.

Recent focus has been given to the fact that the comparison of life expectancy conceals similarities in the age patterns across groups. That is, the fact that the life expectancy of a group (X) is higher than that of another (Y) does not imply that all individuals from group Y will die before all individuals from group X, but rather that on average they will die sooner. At the individual level, the out-survival probability performs all possible pairwise comparisons to estimate the probability that a random individual from group X outlives a random individual from group Y. In this way, it explicitly takes into account the experience of each member of the population [46] (see Supplementary Materials for more details).

In the context of income stratification, [49] proposed a non-parametric approach to measure multi-group stratification. The stratification index for two groups is estimated by comparing the ranks of all individuals in both groups. This can be stated in terms of out-survival probabilities as the out-survival probability between two age-at-death distributions^1^ minus its complement. [49] additionally proposed a multi-group non-parametric index of stratification, which performs all group-pairwise comparisons of ranks of individuals to then estimate the weighted average of the pairwise-group comparisons. More details of these measures are presented in the Supplementary Materials.

At the group level, [38] proposed to use the Tanimoto or Jaccard index to measure lifespan stratification. Specifically, the authors use the complement of the Tanimoto index, which is the proportion of non-overlap of two lifespan distributions (see Sect. 3.1 for more details), to quantify the proportion of non-overlapping area between two age-at-death distributions. Their measure is called *non-overlap index*.

The concept of overlap is new in demography but not in other fields. In ecology, it has been used to study niche overlap [20]. It has also been applied to evaluate targeting methodologies of social programs [9] and in applied psychology [27], among other applications.

[38] presented two theoretical extensions of the non-overlap index for measuring stratification between more than two groups, but barely explored them empirically. In the following section, we apply the pairwise non-overlap index ($S^P$) to different data availability contexts, including cases where individual-level data to partition the population is not readily available (unlike Shi et al.’s empirical investigations). In the main text we only present the results of the $S^{P}$, however in the Supplementary Materials we include the formulas and the results for three other measures of multi-group distributional dissimilarity: total non-overlap index [38], pairwise out-survival probability [46] and non-parametric stratification index [49]. Results for the four measures are indeed very similar among each other (see Supplementary Figs. S11 and S16 and also the Discussion section).

## Methods

### Pairwise non-overlap index

Probability metrics quantify the distance between two statistical objects, such as random variables or samples. For our purposes, we focus on probability distributions, specifically age-at-death distributions derived from a life table. Some examples of probability metrics are the total variation, Kullback–Leibler divergence, Hellinger distance, Tanimoto or Jaccard index, and $\chi ^2$ distance, among others.

The Tanimoto or Jaccard index is a measure of similarity between two probability distributions that has been widely used in different fields, such as ecology, sociology and chemistry. It is the ratio of the overlapping to the total area between the curves of two densities. In our context, let $d^i_x$ denote the life table age-at-death distribution of group *i* at age *x*, and let $\alpha$ and $\omega$ represent the first and last ages in the life table. We further denote by $\varvec{d}^i= \left( d^i_{\alpha },d^i_{\alpha +1}\dots ,d^i_{\omega }\right) '$ the vector of the age-at-death distribution of group *i*. The age-at-death distribution is a proper density, meaning that $\sum _{x=\alpha }^\omega d_x^i = 1$. The Tanimoto or Jaccard index at age $\alpha$ for two populations is calculated as follows: $\tilde{\mathrm{S}}_\alpha (\varvec{d}^1,\varvec{d}^2) = \frac{\sum _{x=\alpha }^\omega \min \{d_x^1, d_x^2\}}{\sum _{x=\alpha }^\omega \max \{d_x^1, d_x^2\}}$, where $0\le \tilde{\mathrm{S}}_\alpha \le 1$. Specifically, $\tilde{\mathrm{S}}_\alpha$ is equal to 1 when $\varvec{d}^1$ and $\varvec{d}^2$ overlap (no inequality), while it is equal to 0 when they do not overlap (maximum inequality).

Given that we are interested in the dissimilarity between two distributions, we follow [38] and use the complement measure, defined as the non-overlap index:

1 $$\begin{aligned} \textrm{S}_\alpha (\varvec{d}^1,\varvec{d}^2) = 1 - \frac{\sum _{x=\alpha }^\omega \min \{d_x^1, d_x^2\}}{\sum _{x=\alpha }^\omega \max \{d_x^1, d_x^2\}} \end{aligned}$$

Using this framework, the first multi-group extension proposed by [38] is given by the weighted mean of the non-overlap index between the age-at-death distribution of all pairs of groups. Formally, the pairwise non-overlap index at age $\alpha$ for *n* population’s subgroups is defined as:

2 $$\begin{aligned} \textrm{S}^P_\alpha = \frac{\sum _{i<j} w_i w_j S_\alpha (\varvec{d}^i,\varvec{d}^j)}{\sum _{i<j} w_i w_j} \end{aligned}$$

where $d^i$ is the life table age-at-death distribution of group *i*, and $w_i$ is the population share of group *i* (where $\sum _{i=1}^n w_i = 1$). Notice that in the case of two groups, the $\textrm{S}$ and the $S^P$ are equivalent.

**Fig. 2:**
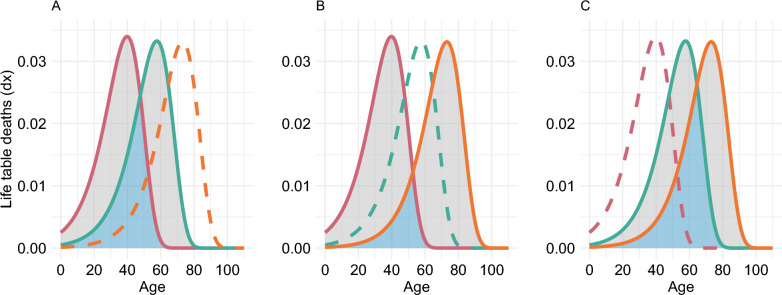
Areas considered in the computation of the pairwise non-overlap index ($S^P$) for three groups. The grey-shaded region is the non-overlapping area between the age-at-death distribution of the two group under comparison (solid lines), while the blue-shaded region is the overlapping area between the same distributions. *Source:* Authors’ own elaborations

For a graphical representation of the areas considered in the computation of the $S^P$, Fig. 2 shows such areas for a hypothetical example with three population’s groups. The non-overlap index between each pair of groups is the ratio of the grey-shaded area to that of the total shaded area (blue and grey). The $S^P$ is estimated as the weighted sum of the non-overlap index from the shaded areas from panels A, B and C.

### Properties

The non-overlap index has some useful properties for the comparison of age-at-death distributions (see [38] for specific details). Given that the $S^P$ is an extension from it, it inherits some of its properties. It is symmetrical, meaning that the result is independent of the order of the groups under comparison. This implies that the $S^P$ can be applied to measure between group mortality inequalities with any population grouping (race, education, area-level indicators, occupation), and not just to ordinal categories such as income level.

The $S^P$ ranges between zero and one. Zero denotes absence of inequality and it is attained if and only if all age-at-death distributions are exactly the same. On the contrary, the maximum value of one is reached when none of the distributions overlap. This is a highly unlikely scenario for human mortality given the restricted domain of the age at death and the regular pattern of mortality. Nonetheless, we found that the non-overlap index between the age-at-death distributions of the Swedish populations in 1773 and 2023 [12] is 0.93, which indicates that it is possible to get high values of the non-overlap index in human populations. However, when comparing subgroups from a single population, we would expect smaller differences in the age-at-death distributions and thus smaller $S^P$. Both limits are unique, and can only be attained in the conditions mentioned before.

The $S^P$ is sensitive to changes in the distribution of all subgroups, not only the extremes. For example, lets assume that the three distributions in Fig. 2 have equal weights. If the middle distribution (blue) were to move closer to either of the other distributions, the $S^P$ would change, the magnitude of the change depends on the specific change of the middle distribution. As for the sensitivity to movements of a single individual, the non-overlap index has the weakness that it is only sensitive to changes across the ages at which the age-at-death distributions intersect [10]. For example, in Fig. 1 an increase in the age-at-death of an individual in the group with the lower life expectancy (pink line) will only cause a decrease in $S^P$ if the increase takes the individual from below the intersection of the curves (61.1 years in panel A and 76.5 years in panel B) to above it.

The $S^P$ does not require a reference distribution nor distributional assumptions, as it measures the distance between all pairwise combinations of distributions. Consequently, the $S^P$ does not assume that all groups should move towards a *best practice* shape with high life expectancy and low lifespan variation. As such, it is possible for two populations with significantly dissimilar internal distributions by group to share the same $S^P$.

The $S^P$ incorporates information on the share of the population in each group through the weights. This has been pointed out as a desirable property in the context where strategies to decrease health inequalities sometimes focus on the social determinants, for example promoting education [32]. Under fixed group-specific mortality, the $S^P$ changes according to the population share in each group (see Supplementary Fig. S2). The direction and magnitude of the change will depend on the dissimilarity between distributions and on the initial weights. Notice that users of the $S^P$ could subjectively *remove* the weights from the formula—if they wished to—by assuming that all groups have equal weights. The assumption underlying this decision is that what is important is the group, regardless of its size [14]. This would be a practical assumption in cases where population weights are unknown.

The $S^P$ is not free of limitations. As noted by [38], the non-overlap index will only be suitable for measuring lifespan stratification when age-at-death distributions can be considered to be hierarchically layered,^2^ which is generally true for human populations. Additionally, it is important to notice that it remains constant under certain transformations in the distributions. Specifically, if all distributions shift horizontally by the same magnitude, then the $S^P$ will not change. This particular insensitivity is a desirable property as changing mortality does not necessarily mean changes in between-group differences.

### Decomposition of subgroups’ contribution

To estimate the marginal contribution of each subgroup to $S^P$ in a given year, country and sex one could apply Shapley decomposition [41]. Although Shapley decomposition has a cooperative game-theory foundation, it has been adopted as an approach to decompose inequality indices [41] into the contribution of each population subgroup or different factor components [4]. In the context of this paper, the Shapley value decomposition estimates the marginal contribution of each sequentially eliminated population subgroup and then estimates the average of its marginal contributions in all possible elimination sequences.

The Shapley value for group *i* is given by

3 $$\begin{aligned} D_i = \sum _{s \subseteq N \backslash \{i\}}\frac{|s|!(n-|s|-1)!}{n!}[S^P(S \cup \{i\})-S^P(S)] \end{aligned}$$

where $D_i$ is the marginal contribution of the *i*-th group, *N* represents the set of all *n* groups, *S* is the subset of *N* without the *i*-th group with $|S|=s$ and, $S^P(Y)$ is the pariwise non-overlap index evaluated in the corresponding set *Y*. The term $\sum _{s \subseteq N \backslash \{i\}}\frac{|s|!(n-|s|-1)!}{n!}$ denotes the probability of randomly selecting set *S* from *N*. This decomposition is additive, meaning that the marginal contribution of each population subgroup adds up to the value of the overall non-overlap index $\sum _i D_i = S^P$. The Shapley value decomposition could also be applied to the other measures of distributional dissimilarity presented in the Supplementary Materials.

### Decomposition of changes over time

The inputs to estimate the $S^P$ are the education-specific mortality rates and the population exposures (or weights) in each group. Then, any change in $S^P$ would be a consequence of a change in either the group-specific mortality levels or the distribution of the population by socioeconomic group. We refer to the second as changes in population composition.

To further understand what drives the changes over time of the $S^P$ and compare them to those of the RII, we decompose both measures into changes in group-specific mortality and changes in the population composition. The decomposition is implemented using the stepwise replacement algorithm proposed by [1] using the *DemoDecomp* R package [33] (see Supplementary Materials for details).

## Empirical applications

In this section, we measure socio-economic inequalities in mortality in two applications with different data requirements: educational groups and groups defined by an area-level deprivation measure. We use available life tables by socioeconomic status to provide some empirical analysis of socioeconomic inequalities in mortality by comparing the $S^P$ with other conventional relative indices: the ratio of life expectancy, the ratio of lifespan variation (measured with the standard deviation of the ages-at-death), and the relative index of inequality (RII) of age-standardized mortality rates. These measures were chosen because of their frequent use in the literature of socioeconomic inequalities in mortality. Comparisons with absolute indices of inequality are presented in the Supplementary Materials. We use the standard deviation of the age-at-death distribution to measure lifespan variation due to its simplicity for interpretation and the fact that it is in the same scale as life expectancy (years). Moreover, measures of lifespan variation are highly correlated with each other [45, 47], so the choice of measure will likely not affect our main conclusions to a great extent.

We start by analysing mortality inequality by educational groups (low, middle, high) in Denmark and Sweden from 1991–1995 to 2011–2015 in Sect. 4.1. We then move to the study of socioeconomic inequality by area-level deprivation index in England from 2006–2008 to 2014–2016 in Sect. 4.2. All of our analyses and results are fully reproducible using the data and codes provided in the open-access repository.

### Mortality inequality by education in Denmark and Sweden

Information on the socioeconomic status of older individuals is limited, even in Nordic countries with high quality register data such as Sweden and Denmark. This limitation is often worked around by restricting the age range in the analysis. [23] developed a non-parametric approach to reconstruct the education-specific composition and mortality curves of the older population. The method redistributes cases with unknown educational attainment and extrapolates the mortality curves and population shares by education level from the last available age-group with complete information on education. Using the estimated mortality values, the authors construct life tables by sex and education level (see [23] for details on the methodology).

We use the life tables by education estimated by [23] for Denmark and Sweden. Abridged life tables, starting from age 30, were estimated by sex for 5-year age groups and 5-year periods from 1991–1995 until 2011–2015 for three education levels. The open-age group is 90 years and above. The Swedish data only covers the population born in Sweden. Education categories are based on the International Standard Classification of Education (ISCED) and are classified as: (i) low (ISCED 1–2, primary and lower secondary education), (ii) middle (ISCED 3–4, upper secondary education) and (iii) high (ISCED 5–6, tertiary education). Additionally, to estimate the exposures of each education group, we obtained the population structure by sex, 5-year age groups and 5-year periods for each country from the Human Mortality Database (HMD) [12] and distribute them according to the weights provided by [23].

**Fig. 3:**
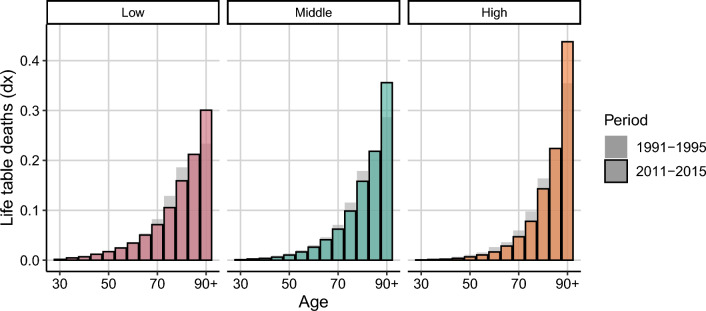
Age-at-death distributions by 5-year age groups for Swedish females by education level in 1991–1995 and 2011–2015, ages 30–90+. Note: Each age group’s value is displayed in the respective age group’s lower limit. *Source:* Authors’ elaborations on data from [12, 23]

We begin by discussing the case of Swedish females. Figure 3 shows the age-at-death distributions for Swedish females by educational level in two points in time, 1991–1995 and 2011–2015. The actual curves do not drop to zero on the right hand side, a pattern often encountered in low mortality countries with high life expectancy when data for the last age groups is aggregated at a not too old age, as in the present example. Regardless of this unusual shape in the age-at-death distribution, the $S^P$ may still be estimated as the distributions still add to one.

Over time, age-at-death distributions for all education groups for Swedish females shifted to older ages. Between 1991–1995 and 2011–2015, life expectancy at age 30 increased from 51.2 to 52.3 for the low educated, and from 54.9 to 56.7 for the high educated. Lifespan variation, measured by the standard deviation of the ages-at-death, increased for the low educated (11.7 to 11.9), while it decreased for the middle and high educated, from 11.2 to 10.7 and from 10.9 to 9.7, respectively. Additionally, during the study period Sweden underwent educational expansion. In 1991–1995, 46 percent of Swedish females had low education and only 17 percent attained high education. In 2011–2015 the low educated reduced to 23 percent and the high educated increased to 32 percent. Similar changes in mortality and in educational expansion are observed for Swedish males and for Danish population during the study period, with life expectancy increases in all the education groups and lifespan variation decreases only in the middle and the high educated groups.

**Fig. 4:**
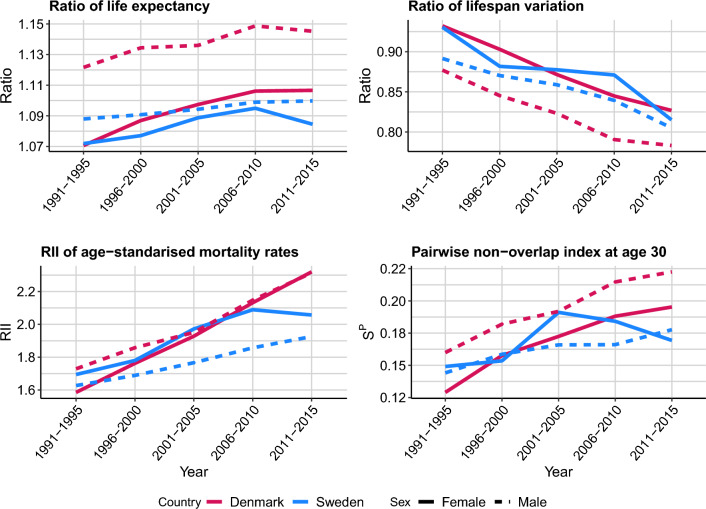
Trends in relative measures of inequality in mortality by sex for population groups defined by education level, Denmark and Sweden, 1991–1995 to 2011–2015. *Source:* Authors’ elaborations on data from [23] and [12]

Figure 4 shows the $S^P$ at age 30 for Sweden and Denmark by sex alongside three commonly used relative measures of socioeconomic inequalities in mortality: the ratio of life expectancy at age 30, the ratio of lifespan variation at age 30 (measured with the standard deviation of the ages-at-death), and the relative index of inequality (RII) of the age-standardised mortality rates (using the WHO World Standard population). Figure S4 in the Supplementary Materials shows the absolute counterparts of these measures. We start by comparing the trends of these measures and then move on to analysing their levels.

For Swedish females, the ratio in life expectancy increased until 2006–2010, and then decreased afterwards. Conversely, the ratio in lifespan variation^3^ increased throughout the study period, though it stagnated between 1996–2000 and 2006–2010. From 1991–1995 to 2006–2010 the ratio in life expectancy and the ratio of lifespan variation had a relative changes of 2.2 percent and $-$12.4 percent, respectively. This difference further increased in the last period. The RII shows more pronounced changes, with a relative change of 23.3 from 1991–1995 to 2006–2010, and a relative change of only 21.4 percent if we consider all the study period. Focusing on the last 5-year period, the inequality measures suggest different conclusions regarding socioeconomic inequalities in mortality: all measures except the ratio in lifespan variation indicate decreasing inequalities, while the ratio in lifespan variation indicates increasing differences in the lifespan variation of the low and the high educated. The $S^P$ started decreasing since 2001–2005, suggesting that equality increased, and that the reduction of inequalities occurred before what the ratio in life expectancy and RII measures suggest. This example shows that patterns of convergence between groups may be hidden when looking only at summary measures of the age-at-death distribution such as the ratio or the gap in life expectancy or lifespan variation. For Danish males and females, the $S^P$ shows similar trends to the ratio in life expectancy and lifespan variation, with all measures reflecting increasing inequalities across groups.

The level of inequality across countries and sexes is dependent on the measure used. For example, Swedish females have the lower level of inequality according to the ratio in life expectancy, but based on the evidence from the RII and the $S^P$ Swedish males do in some years. Regarding the highest level of inequality, Danish males are at the front throughout most of the study period in all measures. The most striking difference in the level of inequality between measures is indeed reflected in Swedish females. According to the ratio of life expectancy, it has the lowest level of inequality throughout most of the periods. However, according to the $S^P$ and RII, it had the highest level of inequality amongst the studied populations in 2001–2005. This suggest that the $S^P$ is incorporating information that is not reflected in the other inequality measures.

Supplementary Fig. S5 shows the Shapley decomposition of $S^P$. As expected, the low and the high education groups have the highest marginal contributions while the middle group has the lowest. Interestingly, Danish females are the only case in which the low educated have the highest marginal contribution. The pairwise non-overlap index (see Fig. S6 in the Supplementary Materials) suggests that this might be because of a greater similarity between the middle and high educated than the middle and low educated, the latter being the trend in all other cases. This suggests that the lower educated Danish females are lagging behind in terms of mortality more than lower educated groups in the other populations considered.

**Table 2:** Decomposition of the change in the $S^P$ between 1991–1995 and 2011–2015 by sex, Denmark and Sweden

	Sweden		Denmark	
	Female	Males	Female	Males
Population composition	0.0044	0.0045	− 0.0089	0.0048
Mortality	0.0158	0.0290	0.0752	0.0578
Low	− 0.0546	− 0.0576	− 0.0586	− 0.0502
Middle	− 0.0061	− 0.0085	0.0255	0.0002
High	0.0765	0.0951	0.1080	0.1080
Total change	0.0202	0.0335	0.0663	0.0626

Finally, the age-specific decomposition of the changes in the $S^P$ between 1991–1995 and 2011–2015 (see Fig. S7 in the Supplementary Materials) reveals that for both countries and sexes, increases in the $S^P$ were primarily driven by mortality changes (Table 2). The low educated group contributed to the decrease in inequality, the high educated group had the opposite contribution. Changes in the population composition contributed to the increase the $S^P$ in most cases, with the exception of Danish females.

### Mortality inequality by area-level deprivation index in England

Analysis of socioeconomic inequalities in mortality is often restricted to countries with high quality individual-level data, which allow to derive unbiased mortality estimates by population’s subgroups [15, 40]. Such data is available for a few countries and can suffer from issues such as increasing selection of some categories and changing composition [21]. In contexts where individual-level indicators of socioeconomic status are not available, it is possible to estimate area-based mortality indicators [7, 37]. These have the added advantage of providing estimates for the whole population, starting from age zero rather than from an older age. Given that area-based life tables can be derived, it is possible to estimate the $S^P$ to measure inequality in mortality.

Here we present an example using the English Index of Multiple Deprivation (IMD) 2015 deciles [42]. The IMD is the official measure of relative deprivation for small areas in England. It combines information from seven domains: income, employment, education, health, barriers to housing and services, crime and living environment. It ranks 32,844 small areas, with roughly the same population, by level of deprivation. The areas are then grouped into deciles, with each decile containing 10 percent of the small areas. We use the life tables by IMD decile estimated for England by UK’s Office for National Statistics [24]. These are single-age life tables by sex starting at age zero. The estimates are based on mortality rates calculated for a three year periods from 2006–2008 until 2014–2016. Mid-year population estimates by age, sex and deprivation deciles from the [25] are used to derive the share of population in each decile.

**Fig. 5:**
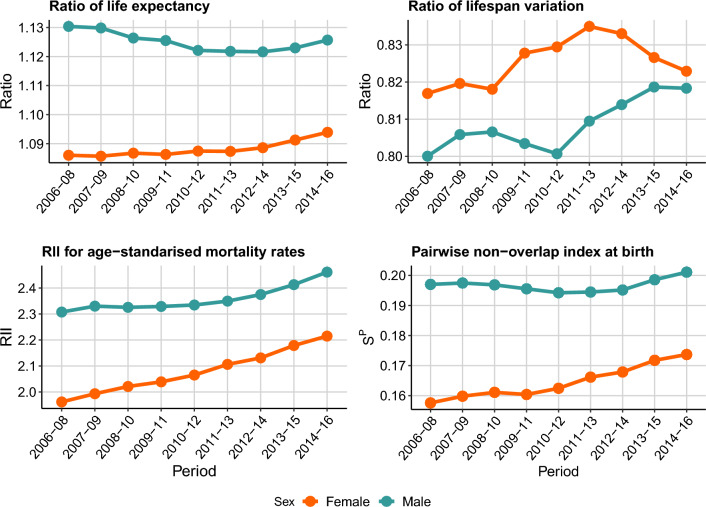
Trends in relative measures of inequality in mortality by sex for population groups defined by area-level deprivation deciles, England, 2006–2008 to 2014–2016. *Source:* Authors’ elaborations on data from [24, 25]

Figure 5 presents the $S^P$ for groups defined by deprivation deciles in England for the period 2006–2008 to 2014–2016 by sex alongside the same three relative measures of socioeconomic inequalities in mortality used in the previous example. In this case the life expectancy and the lifespan variation are calculated at birth. Figure S12 in the Supplementary Materials shows the absolute counterparts of these measures. Again we start by discussing trends and then levels of inequality.

For females, the $S^P$ steadily increased during the study period (relative increase of 10.2 percent). This trend is similar to the one portrayed by the RII (relative increase of 12.9 percent), but different to that from the ratio of lifespan disparity, which shows a decrease followed by an increase in more recent years, respectively. Conversely, the ratio of life expectancy shows an almost constant trend, detecting no change in inequalities between deciles, with a relative change of 0.1 percent during the studied period.

In the case of males, both trends, ratio of life expectancy and $S^P$, show similar patterns, with a slow decrease in the first years followed by an increase in the later years. However, the direction of the relative change from 2006–2008 to 2014–2016, differs by measure. According to the ratio in life expectancy, there was a relative decrease of 0.1 percent in inequality, while the $S^P$ points to a relative increase of 2.1 percent. Conversely, the RII increases throughout the whole study period. Its relative change was of 6.2 percent for the whole period. The ratio of lifespan variation decreased over time, with small fluctuations in certain periods.

All four measures of inequality show similar results when comparing the levels of inequality by sex. Males have higher inequality in mortality than females, with the gap reducing by the end of the study period. The most notable reduction in the difference in inequality levels by sex is observed in the ratio of lifespan variation with a relative change in the gap between sexes of $-$73.7 percent. Compared to the RII, the $S^P$ shows a greater reduction in the gap in the level of inequality between both sexes, with a relative change of $-$30.5 percent compared to $-$28.8 percent for the gap in RII.

The Shapley value decomposition (see Supplementary Fig. S13) shows that the lower decile has the highest marginal contribution to the $S^P$ throughout the study period, suggesting that the age-at-death distribution of this group is lagging behind all others. The middle deciles (5 and 6) have negative Shapley values, meaning that if one were to remove these groups, the $S^P$ would increase.

From the stepwise decomposition (see Fig. S14 in the Supplementary Materials), we see that the increase in the $S^P$ occurred because changes in mortality of the highest deciles offset the contributions of the lowest deciles. Additionally, we see that for women contributions are concentrated in a narrower age-range than for men. The contribution of the change in population composition are small compared to those of the change in mortality. This is expected, as each decile contain roughly 10 percent of the population and it is fairly stable over time.

## Discussion

The aim of this paper was to rigorously assess and further implement the multi-population extension of the recently proposed statistical distance metric to measure distributional dissimilarity in mortality and to evaluate whether using the information contained in the age-at-death-distribution and the mortality experience of all groups provides new and additional insights on socioeconomic inequalities in mortality than conventional summary-based measures. For this purpose, we used the the pairwise non-overlap index ($S^P$) proposed by [38], that captures distributional differences between two or more population’s subgroups.

For the two examples shown in this paper, changes in the $S^P$ for all the study periods are mainly driven by mortality changes in all subgroups. Higher educated groups and higher deciles contribute to increasing inequalities, while lower educated groups and lower deciles contribute to decreasing inequalities. In both examples the $S^P$ increased over time, suggesting that mortality of the higher SES is improving faster than that of the lower SES groups, thus increasing inequalities.

Similar to Shi and colleagues ([38]), we find that the $S^P$ can show different pattern from those shown by measures based either on life expectancy or lifespan variation, as in the case of Swedish females between 2006–2010 and 2011–2015. One possible explanation of the different trends is that the $S^P$ reflects both shifts and compression in the age-at-death distributions. During this period, the underlying distribution of high-educated Swedish females had an increase in mortality between ages 80 and 89, which caused a decrease in life expectancy, a sharp decrease in the lifespan variation, and slight increase in its age-standardized mortality rate, reflected in the trends shown in Fig. 4. The age-at-death distribution of the high educated moved closer to those of the other groups, which was captured by the decrease in the $S^P$. In fact, the decomposition of the change in the $S^P$ for this last period shows a negative contribution of the high educated groups to the $S^P$ (Fig. S9 in Supplementary Materials). In the case study of England, during the study period, the age-at-death distributions of all deciles are shifting to older ages and are compressing at older ages. However the lower deciles are doing so at a lower speed than the higher deciles, which results in increasing inequalities over time.

In other cases, for example that of Danish males, the trends in the $S^P$ may not differ from those captured by other measures of inequality. However, as seen in that example, the level of the inequalities reflected by each measure may still be different. Regardless of the case, we believe that the $S^P$ is informative because it captures additional information on the changes of the age-at-death distributions.

The data requirements of the $S^P$ are the same as the ones needed to estimate life expectancy and lifespan variation by socioeconomic groups or any other population grouping (by race, gender, over time, etc.), as it is estimated from the life table death distribution. To illustrate this, we presented an application on how the $S^P$ may be used for area-level socioeconomic measures, which may be available in contexts where individual-level data on socioeconomic position is not available. We hope that this measure encourages more statistical offices to produce life tables by different socioeconomic characteristics.

As mentioned in Sect. 2, besides the $S^P$ there are other measures to estimate dissimilarity between age-at-death distributions. Figures S11 and S16 in the Supplementary Materials show different distributional dissimilarity measures for the empirical examples presented here. With the exception of the total non-overlap index ($S^T$), all other measures show very similar patterns. Ultimately, the choice of measure will depend on the context and the purpose of the analysis. For example, if one were interested in the probability that an individual from a group with lower life expectancy outlives an individual from a group with higher life expectancy, one might use the multi-group extension of the out-survival probability.

Shi et al. [38] propose two multi-group extensions of the non-overlap index, one performing all pairwise comparisons and another comparing each group’s distribution to a reference distribution. We found that when the reference distribution is the “population average”, the theoretical maximum value of the *total non-overlap index* ($S^{T}$) depends on the number of groups and on the population share of each group. As such, we have derived a normalization constant to ensure that the maximum value of one is always attained also in this case (see Supplementary Materials).

All measures of inequality use the age-at death distribution as input and end up with a number summarizing the comparison. Those that are based on summary measures, first summarize the distribution into a single number –be it age-standarized mortality rates, life expectancy or lifespan variation– and then compare this summary quantity. Conversely, the non-overlap index compares the age-at-death distributions across all ages and then summarizes such comparison. The advantage of the second approach is that, when decomposed, all age-groups receive the same weight and therefore it is easier to identify where mortality differences stem from.

Recent developments have focused on decomposing the total lifespan inequality of a population into between- and within-group contributions [28]. The $S^P$ goes beyond this line of research as it is not a measure of overall lifespan inequality, but rather of the overall dissimilarity across multiple distributions. It could be pointed out that, although our main goal is not to measure total lifespan inequality, the $S^P$ implicitly accounts for this by comparing the full age-at-death distributions; as such, it is an indicator of inter-group inequalities, while taking into account inter-individual inequalities. Additionally, it should be explicitly mentioned that this distributional approach supports the argument that in health inequalities, what is morally significant is the systematic association between health and socioeconomic status [2].

The $S^P$ has the potential limitations that it does not indicate the direction of the distributional convergence. For example, if the $S^P$ decreases, it is not possible to distinguish if it is because mortality decreased for the worst-off group or because it increased for the better-off group. The results presented here have two limitations that apply more generally. First, both examples are based on adjusted data, thus the results might be an artifact of the adjustment procedure. However, such adjustment would affect all measures under comparison as they use the same data as input. Furthermore, most data sources of mortality undergo correction and adjustment procedures, so such limitation extends beyond our research. Second, policy interventions are typically based on real populations rather than on synthetic cohorts of the life tables [6]. As the $S^P$ is estimated from life table age-at-death distributions, it might not directly highlight where policy efforts should be primarily concentrated. Nonetheless, we believe that the $S^P$ can still inform policies by complementing the information provided by other measures of inequalities, and by indicating the relevant age-groups of the group-specific life table populations where mortality differences occur.

## Conclusions

In this paper we evaluated the pairwise non-overlap index ($S^P$), a measure of overall similarity across distributions for multiple groups. The $S^P$ can be considered as an alternative measure of inequality in mortality that goes beyond the first two summary measures of the distribution (life expectancy and lifespan variation). Our results show that levels and trends in mortality inequality differ when using the $S^P$ as compared to conventional inequality measures. Compared to the range and the rate ratio, the $S^P$ captures more than just the inequality between the extremes, and compared to the SII and the RII—which take into account all groups in establishing inequality—the $S^P$ does not necessitate of hierarchical categories.

We believe that the measurement of socioeconomic inequalities in mortality could be complemented by measuring dissimilarity between age-at death distributions, and hope that this article contributes to the case of using the age-at-death distributions when comparing population subgroups. This is particularly valuable in a context where mortality improvements are becoming less homogeneous between populations and changes may not be reflected by summary measures. The method we propose can be applied to other data sets, data contexts or research questions, as long as mortality rates by socioeconomic-groups (and potentially also population weights) are available.

## Supplementary Information

Supplementary file 1.

## Data Availability

The original data sets were obtained from ONS and from the authors of [23], these cannot be shared by us. Please contact the authors for obtaining the data; aggregate results from our analysis are available in the Results folder of the open-access repository. The ONS data can be downloaded from the following links: deaths and exposures and life tables.

## Abbreviations

RII: Relative index of inequality
SII: Slope index of inequality

## Footnotes

1 In the discrete case, this is empirically different to the out-survival probability proposed by [46] due to assumptions on the incorporation of deaths that occur at the same age for both groups.

2 In life table notation, two age-at-death distributions can be viewed as hierarchically layered when *x* and $\frac{\{d^i_x-d^j_x\}}{\{d^i_x+d^j_x\}}$ are strongly correlated.

3 Notice that lifespan variation is higher for the low than for the high educated, thus the negative slope. Any deviation from 1 indicates increasing inequalities.
