# Supplementary material for: Reassessing socioeconomic inequalities in mortality via distributional similarities
Source: Popul Health Metr. 2025 Feb 22;23:7. doi: 10.1186/s12963-025-00365-1 (PMC11847365; doi:10.1186/s12963-025-00365-1)
Supplement: Supplementary file 1 [file 12963_2025_365_MOESM1_ESM.pdf]

Supplementary materials of: Reassessing socioeconomic inequalities in mortality via distributional similarities

Ana C. Gómez Ugarte[1], Ugofilippo Basellini[1], Carlo G. Camarda[2], Fanny Janssen[3,4], and Emilio Zagheni[1]

[1] *Max Planck Institute for Demographic Research, Rostock, Germany*
[2] *Institut national d'études démographiques, Aubervilliers, France*
[3] *Aging and Longevity, Netherlands Interdisciplinary Demographic Institute - KNAW/University of Groningen, The Hage, The Netherlands*
[4] *Population Research Centre, Faculty of Spatial Sciences, University of Groningen, Groningen, The Netherlands*

February 17, 2025

In these Supplementary Materials, we provide details on other measures of multi-group distributional dissimilarities. Additionally, we include the absolute counterparts of the results shown in the main text and other supplementary graphs. We also present the detailed explanation of the stepwise decomposition procedure.

Definition and properties

Figure S1 is the absolute counterpart of Figure 1 in the main text. It shows four hypothetical populations along with their respective level of socioeconomic inequality in mortality derived from different absolute measures.

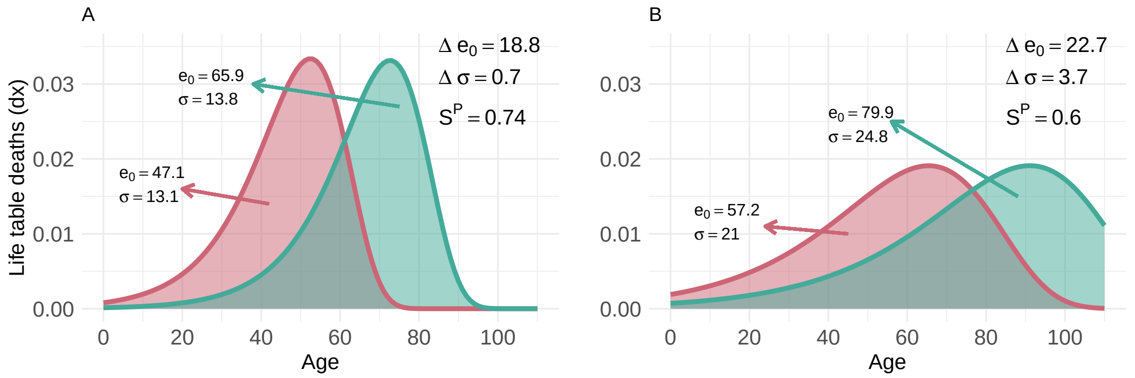

Figure S1: Hypothetical scenarios of the age-at-death distributions of two equal-sized (Panels A and B) or three equal-sized (Panels C and D) population's subgroups.
Note: The black line represents the age-at-death distribution of the total population. The annotations on the graphs include: life expectancy at birth (e_0), lifespan variation (measured by the standard deviation of the ages-at-death, σ), range in life expectancy at birth between both distributions (Δe_0), range in standard deviation of the ages-at-death between both distributions ($\Delta\sigma$) and pairwise non-overlap index (S^P).
Source: Authors' own elaborations.

Figure S2 shows how under fixed group-specific mortality rates, the S^P changes according to the population share in each group. Using the three subgroups from Figure 2 in the manuscript, we simulate different population shares for the groups and estimate the corresponding S^P. In this case, the x-axis represents the weight corresponding to the worst-off group, that with higher mortality (pink curve). The different colors of the curves correspond to the population share in the middle group (blue curve), while the population share of the better-off group (orange curve) is one minus the sum of the weights of the other two groups.

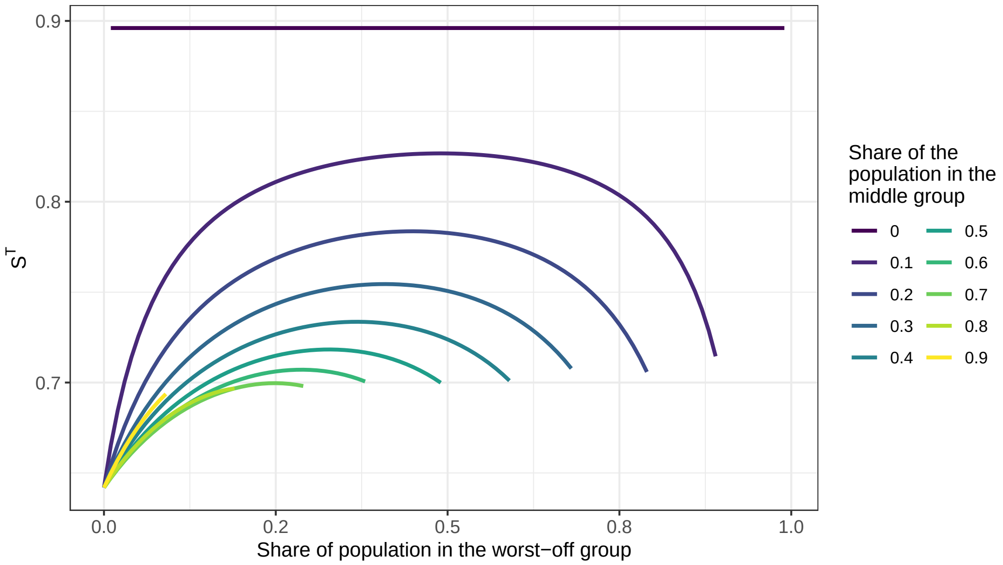

Figure S2: S^P values for simulated weights of the three subgroups from Figure 2 in the manuscript.

Measures of multi-group distributional dissimilarity

Let d_x^i denote the life table age-at-death distribution and l_x^i the number of survivors of group i at age x, and let α and ω represent the first and last ages in the life table. We further denote by $\boldsymbol{d}^i = \left(d_\alpha^i, d_{\alpha+1}^i \ldots, d_\omega^i\right)'$ the vector of the age-at-death distribution of group i.

Total non-overlap index

The second multi-group extension of the non-overlap index proposed by Shi et al. (2023), which we call total non-overlap index is given by:

$$\mathrm{S}_\alpha^T = \sum_i w_i S_\alpha(\boldsymbol{d}^i, \boldsymbol{d}^T) * \frac{1}{\sum_i w_i \left[1 - \frac{w_i}{1+\sum_{j \neq i} w_j}\right]} \tag{1}$$

where d^T is the age-at-death distribution of the total population. It is worth noting that Eq. (1) differs from the formula proposed by Shi et al. (2023) by the term $\frac{1}{\sum_i w_i \left[1-\frac{w_i}{1+\sum_{i \neq j} w_j}\right]}$. We included this term to ensure that S^T can vary between 0 and 1, as the maximum value of the Shi et al. (2023) formula depends on the number of groups n and on the size of the groups.

To estimate d^T one could estimate the mortality rate of the total population as the arithmetic mean of the group-specific mortality rates weighted by the exposures (Feehan and Wrigley-Field, 2021) and then derive the corresponding age-at-death distribution using standard life table techniques. Notice that even in the case of two groups, the S and the S^T are different, as in the first case the dissimilarity is estimated between the two populations, and in the latter case it involves the age-at-death distribution of the total population as well as the group-specific weights.

For a graphical representation of the areas considered in the computation of the total non-overlap index (S^T), Figure S3 shows such areas for the distributions presented in the left panel of Figure S1 and another hypothetical example with three population's groups. The non-overlap index between each group (coloured lines) and the total population (dashed-black line) is the ratio of the grey-shaded area to that of the total shaded area (blue and grey). For the case of two groups (upper panels) the S^T is the weighted sum of the non-overlap index estimated from the shaded areas from panels A and B. For three groups (lower panels), the S^T is estimated as the weighted sum of the non-overlap index from the shaded areas from panels C, D and E.

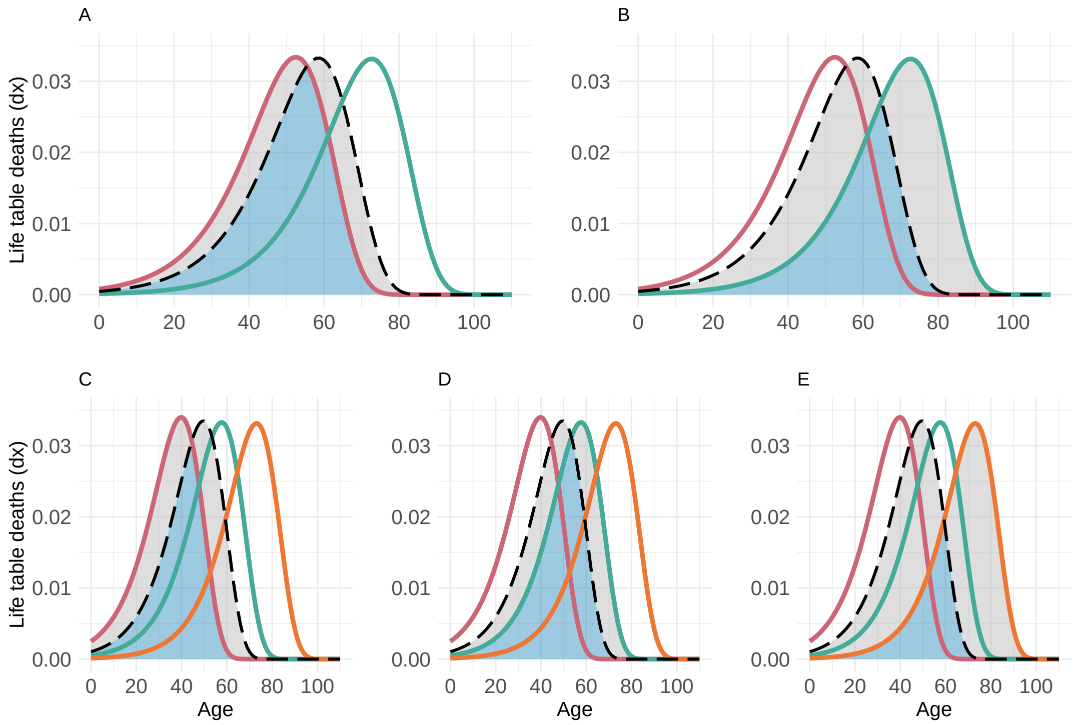

Figure S3: Areas considered in the computation of the total non-overlap index (S^T) for two groups (upper graphs) and for three groups (lower graphs). The grey-shaded region is the non-overlapping area between the age-at-death distribution of the total population (black line) and that of each group (in color), while the blue-shaded region is the overlapping area between the same distributions.
Source: Authors' own elaborations.

Out-survival probability

Vaupel et al. (2021) proposed the out-survival probability to estimate the probability that a random individual from group j outlives a random individual from group i. Let e^i_x be the life expectancy of group i at age x. Then, given that $e^j_\alpha > e^i_\alpha$ the out-survival probability between the age-at-death distributions of groups i and j in the discrete case is given by:

$$OV_\alpha(\boldsymbol{d}^i, \boldsymbol{d}^j) = \sum_{x=\alpha}^{\omega-1} \sum_{y=\alpha+1}^{\omega} d^i_x l^j_y + \frac{\sum_{x=\alpha}^{\omega} d^i_x + d^j_y}{2}. \tag{2}$$

The condition that $e^j_\alpha > e^i_\alpha$ ensures that the out-survival probability is measured from the group with higher life expectancy to the group of lower life expectancy. Thus the out-survival probability measures the probability that a random individual from the group with higher life expectancy (j) out-survives and individual with the group with lower life expectancy (i). It is important to notice that the second term in Equation (2) assumes that for a given age x half of the total deaths of both groups at age x occur first for one group than for the other. That is, only half of the deaths at age x contribute to the out-survival probability. Finally, it should be mentioned that here we ordered the groups by life expectancy, but it is not a necessary condition, one can calculate the out-survival probability between two groups regardless of the *order*.

Following the same logic of the pairwise non-overlap index and the idea of the multi-group stratification index proposed by Zhou (2012), we define the pairwise out-survival probability as the weighted average of all pairwise out-survival probabilities conditional on $e^n_\alpha > e^{n-1}_\alpha > ... > e^1_\alpha$:

$$OV_{\alpha}^{P} = \frac{\sum_{i<j} w_i w_j OV_{\alpha}(\boldsymbol{d}^i, \boldsymbol{d}^j)}{\sum_{i<j} w_i w_j} \tag{3}$$

where d^i is the life table age-at-death distribution of group i, and w_i is the population share of group i (where $\sum_{i=1}^{n} w_i = 1$).

Non-parametric stratification index

In the context of income stratification Zhou (2012) proposed a non-parametric approach to measure multi-group stratification. Here we present the measure translated in terms of age-at-death distributions and life table notation. Let x_k and i be the age and the group of the k-th individual, and e_{α}^{i} be the life expectancy of group i at an initial age α. Following Zhou and Wodtke (2019), the multi-group non-parametric index can be expressed as:

$$SI_{\alpha} = P[x_k > x_l | e_{\alpha}^{k} > e_{\alpha}^{l}] - P[x_k < x_l | e_{\alpha}^{k} > e_{\alpha}^{l}] \tag{4}$$

where $e_{\alpha}^{k} > e_{\alpha}^{l}$ indicates that group k has a higher life expectancy at age α than group l. The index estimates the conditional probability that a random individual from a group of higher life expectancy has a longer lifespan than a random individual from a group with lower life expectancy minus the conditional probability that a random individual from a group of higher life expectancy has a shorter lifespan than a random individual from a group with lower life expectancy. Following Zhou and Wodtke (2019)'s interpretation, the index is an increasing function of the accuracy with which the relative ranking of group-specific life expectancy can predict the relative ranking of individual lifespans. Here we use life expectancy to rank groups by mortality level, but other measures like the modal age at death can be used for this purpose. See Zhou (2012) for other representations of the stratification index.

It is important to notice that the SI does not incorporate the population shares of each group (w_i).

As far as we understand, the out-survival probability is conceptually equivalent to the *Pagree* term in Zhou (2012). The difference between both equations is on the assumption about the contribution to the measure of the deaths that occur at a same age x for both groups under comparison. In the discrete case the out-survival probability assumes that for a given age x half of the total deaths of both groups at age x occur first for one group than for the other. That is, only half of the deaths at age x contribute to the out-survival probability. Whereas the stratification index proposed by Zhou (2012) assumes no contribution from deaths that occur at the same age x in both groups, as their rankings would be the same.

Empirical results

Figure S4 is the absolute counterpart of Figure 4 in the main text. It shows the S^P at age 30 for Sweden and Denmark by sex alongside three commonly used absolute measures of socioeconomic inequalities in mortality: the range of life expectancy at age 30, the range of lifespan variation at age 30 (measured with the standard deviation of the ages-at-death), and the slope index of inequality (SII) of the age-standarized mortality rates (using the WHO World Standard population).

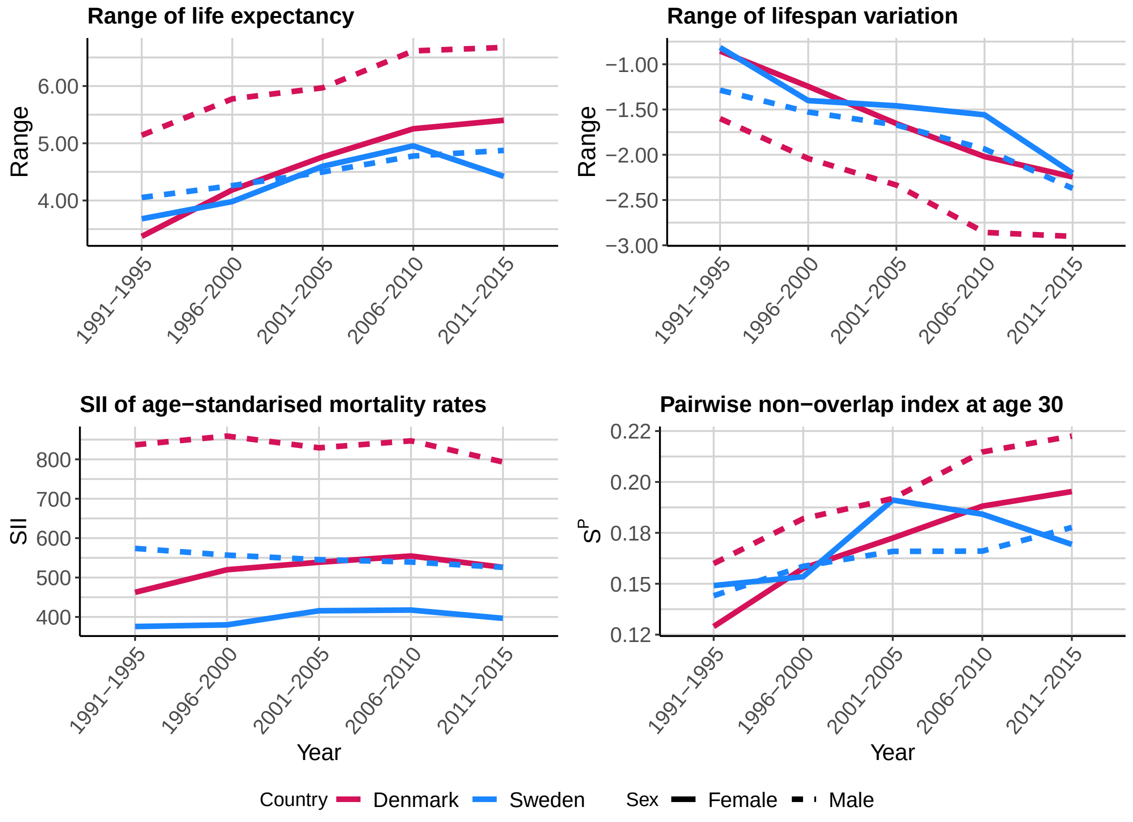

Figure S4: Trends in measures of inequality in mortality by sex for population groups defined by education level, Denmark and Sweden, 1991-1995 to 2011-2015.
Source: Authors' elaborations on data from Németh et al. (2021) and Human Mortality Database (2023).

Figure S5 shows the results of the Shapley value decomposition of S^P.

Figure S6 shows the non-overlap index between each pair of education groups at age 30 for Sweden and Denmark by sex. These are the elements that are weighted to estimate S^P.

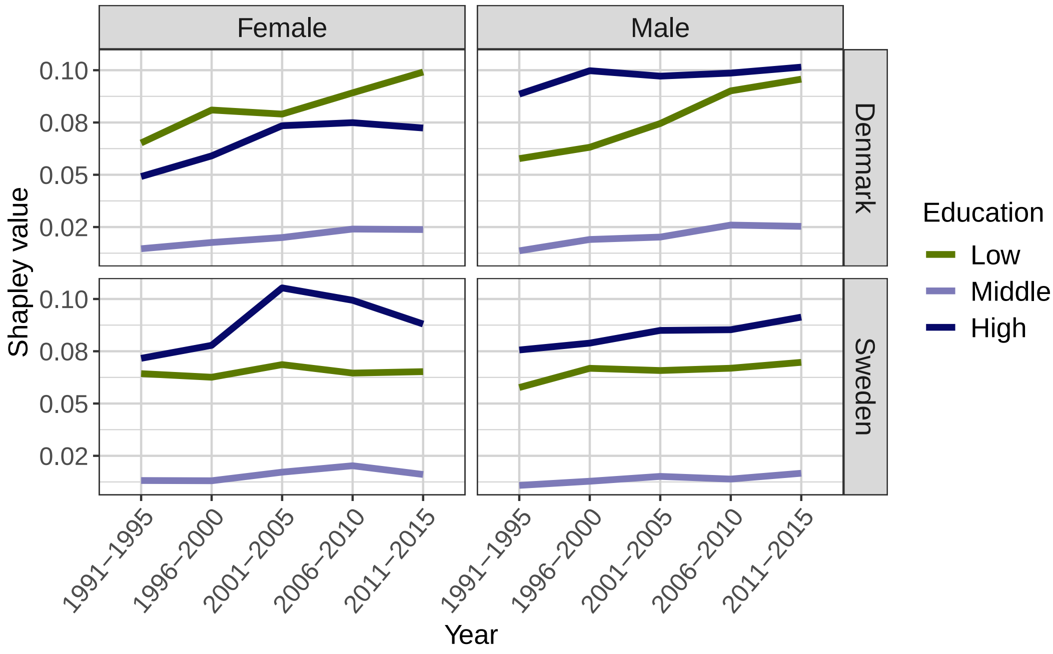

Figure S5: Shapley values of the pairwise non-overlap index (S^P) by education groups for Denmark and Sweden, 1991-1995 to 2011-2015.
Source: Authors' elaborations on data from Németh et al. (2021) and Human Mortality Database (2023).

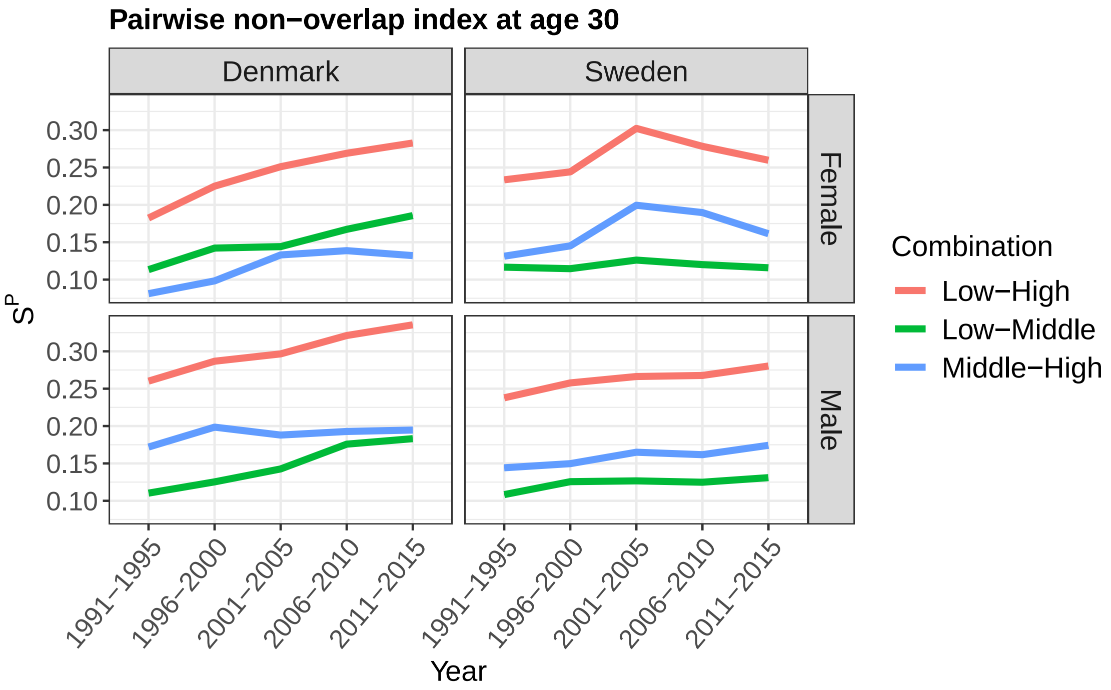

Figure S6: Trends in measures of inequality in mortality by sex for population groups defined by education level, Denmark and Sweden, 1991-1995 to 2011-2015.
Source: Authors' elaborations on data from Németh et al. (2021) and Human Mortality Database (2023).

Figure S7 shows the results of the decomposition of the changes in the S^P between 1991-1995 and 2011-2015 for Sweden and Denmark. The decomposition distinguishes between two separate components: mortality changes versus changes in the composition

of the population. The first can be further decompose into the changes of the mortality of each group.

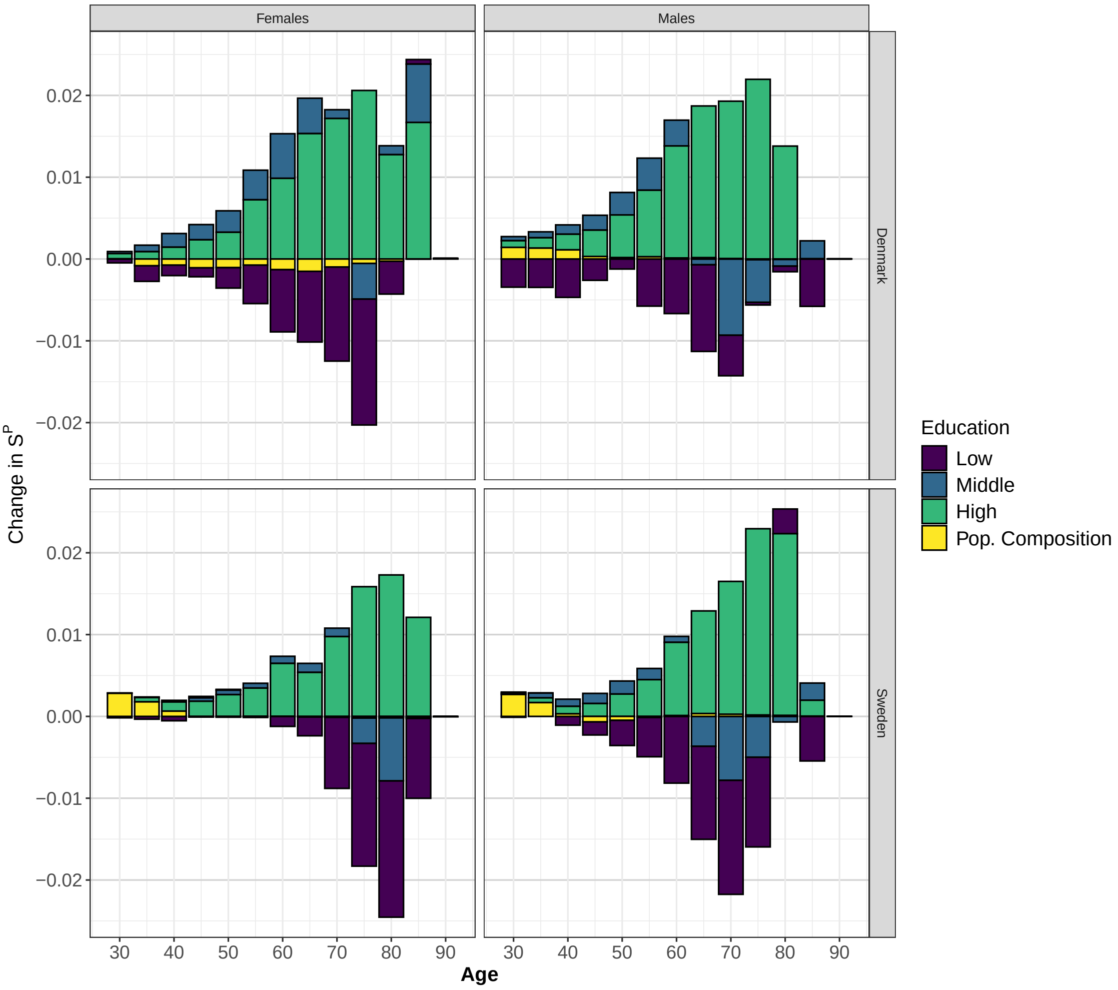

Figure S7: Decomposition by age and education of the changes in the S^P between 1991-1995 and 2011-2015 for Sweden and Denmark.
Source: Authors' elaborations on data from Németh et al. (2021) and Human Mortality Database (2023).

Figure S8 shows the results of the decomposition of the changes in the S^P between 2006-2010 and 2011-2015 for Sweden and Denmark. We include this figure to show how the mortality of the high educated Swedish females contributed to decrease the S^P in the given period.

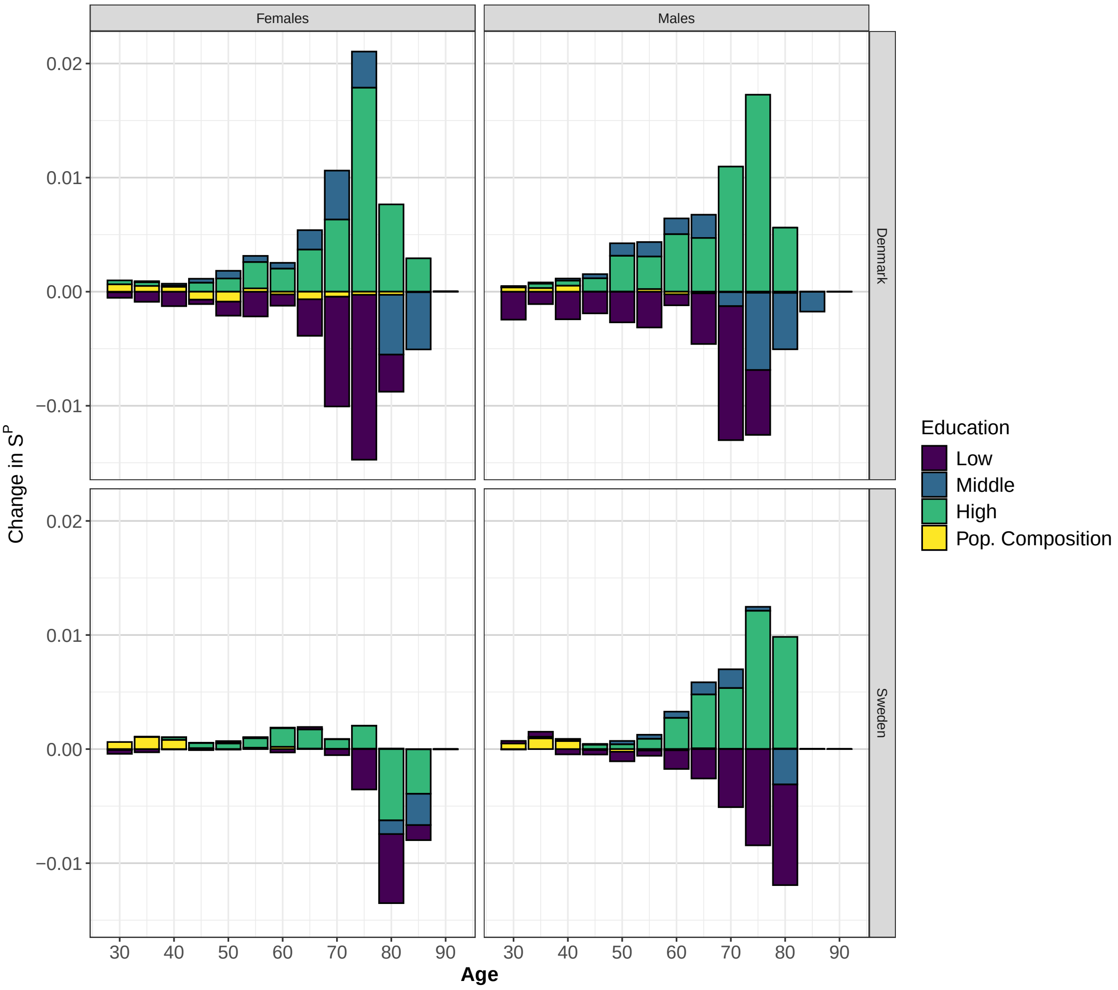

Figure S8: Decomposition by age and education of the changes in the S^P between 2006-2010 and 2011-2015 for Sweden and Denmark.
Source: Authors' elaborations on data from Németh et al. (2021) and Human Mortality Database (2023).

Figure S9 shows the results of the decomposition of the changes in the RII between 1991-1995 and 2011-2015 for Sweden and Denmark. Same as for the S^P, the decomposition distinguishes between two separate components: mortality changes versus changes in the composition of the population.

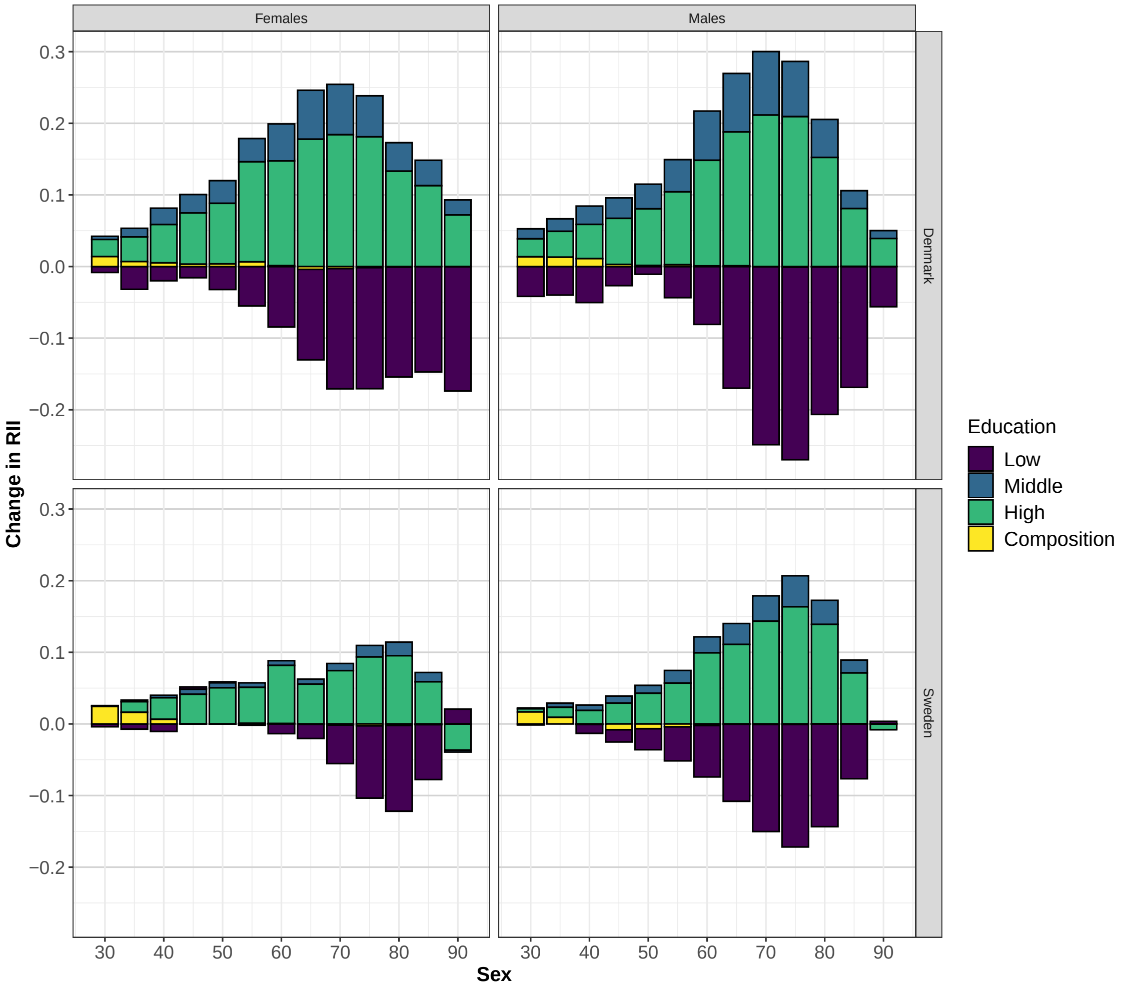

Figure S9: Decomposition by age and education of the changes in the RII of the age-standardized mortality rates between 1991-1995 and 2011-2015 for Sweden and Denmark. *Source:* Authors' elaborations on data from Németh et al. (2021) and Human Mortality Database (2023).

Figure S10 shows the proportion of population by education in 1991-1995 and 2011-2015 for Sweden and Denmark.

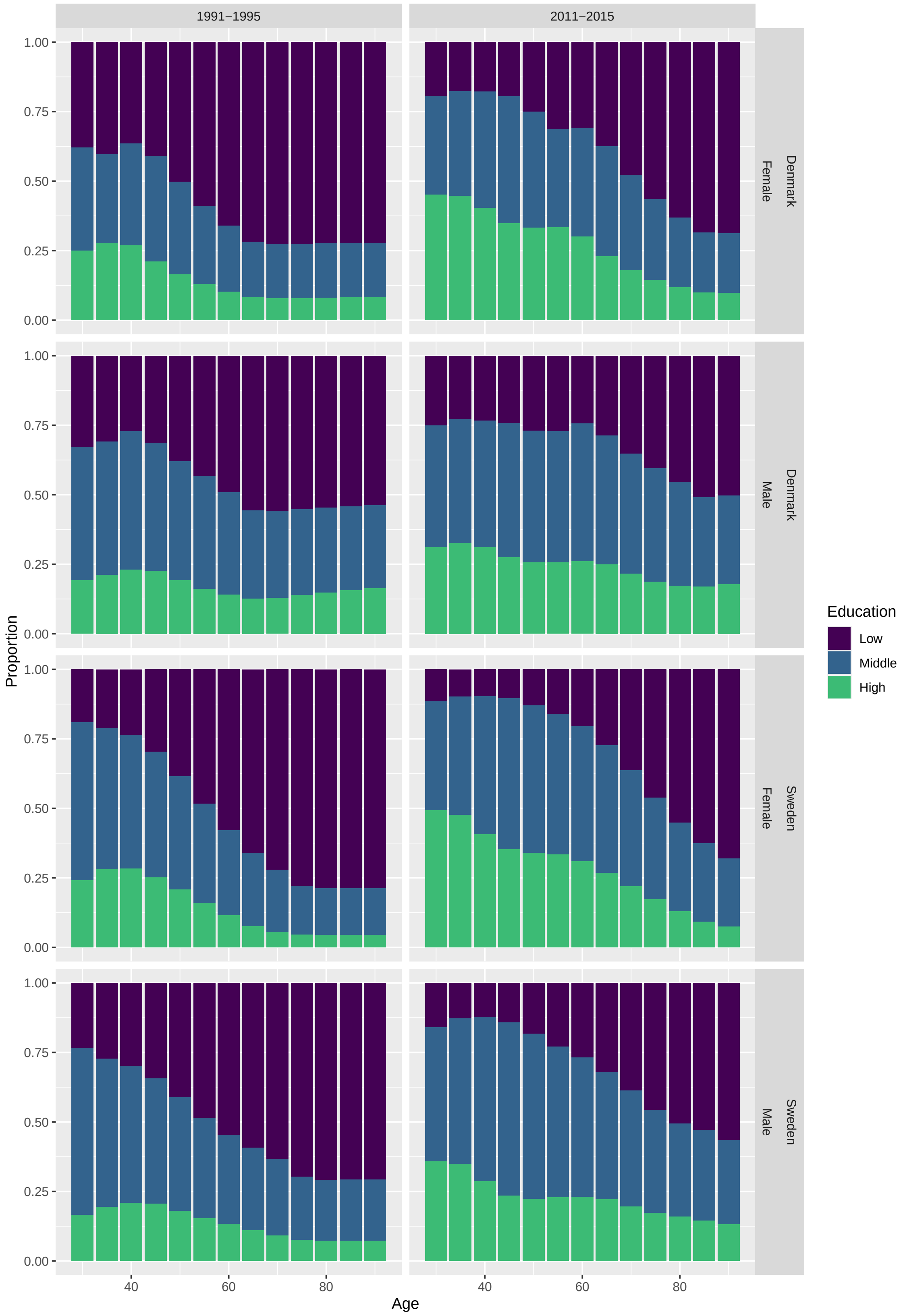

Figure S10: Proportion of population by education in 1991-1995 and 2011-2015 for Sweden and Denmark.
Source: Authors' elaborations on data from Németh et al. (2021) and Human Mortality Database (2023).

Figure S11 shows the trends in four multi-group measures of distributional dissimilarity: pairwise non-overlap index (S^P), total non-overlap index (S^T), stratification index

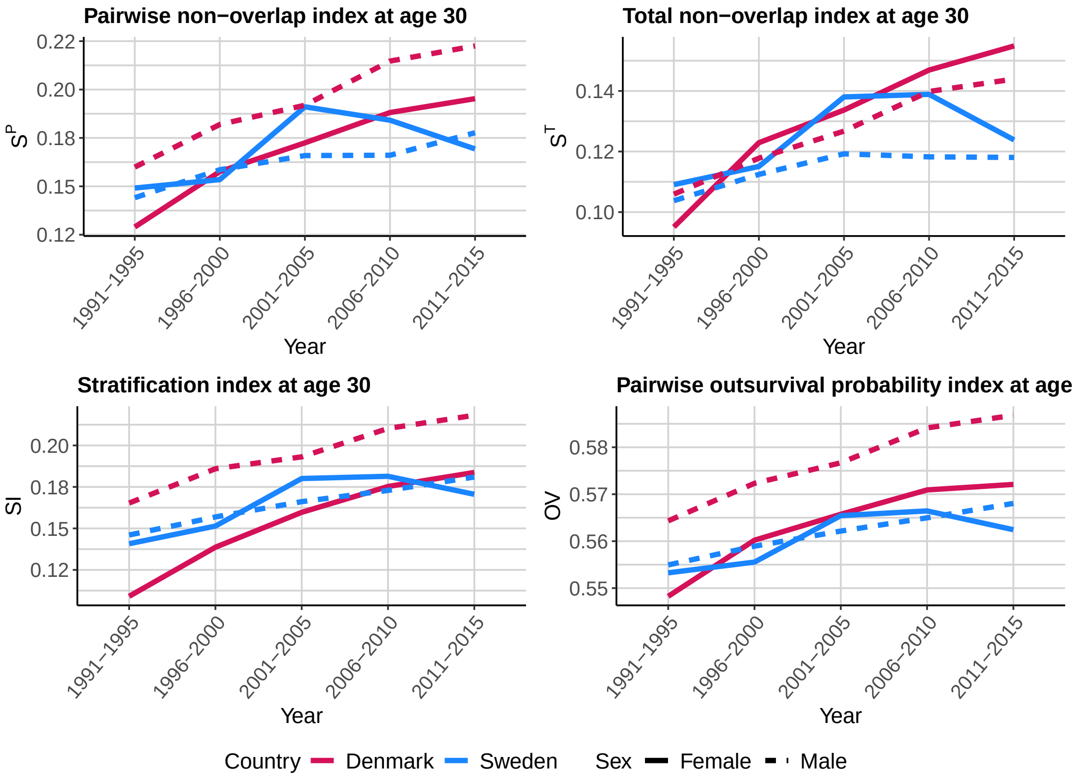

Figure S11: Trends in measures of multi-group distributional dissimilarity in mortality by sex for population groups defined by education level, Denmark and Sweden, 1991-1995 to 2011-2015.
Source: Authors' elaborations on data from Németh et al. (2021) and Human Mortality Database (2023).

(SI) and pairwise out-survival probability index (OV) for the example by education groups presented in the main text.

Figure S12 is the absolute counterpart of Figure 5 in the main text. It presents the S^P for groups defined by deprivation deciles in England for the period 2006-2008 to 2014-2016 by sex. Alongside we include three absolute measures of socioeconomic inequalities in mortality: the range of life expectancy at birth, the range of lifespan variation at birth (measured with the standard deviation of the ages-at-death), and the slope index of inequality (SII) of the age-standarized mortality rates (using the WHO World Standard population).

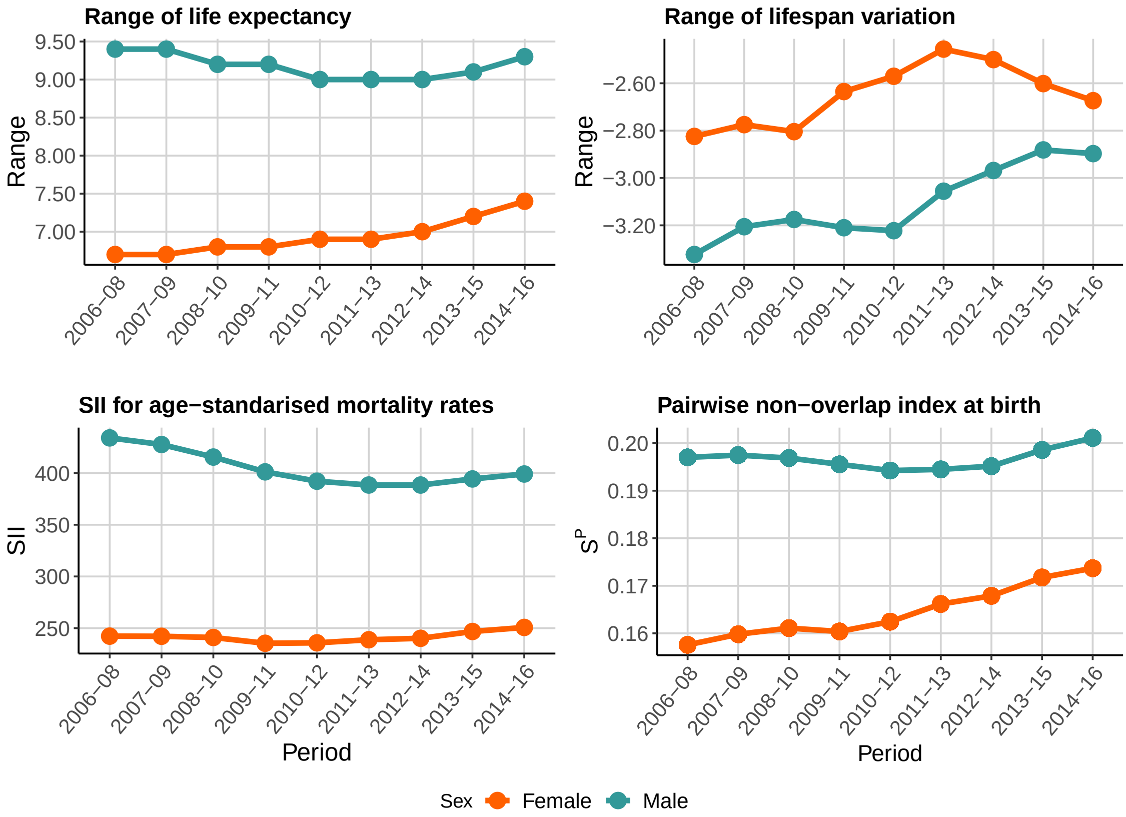

Figure S12: Trends in measures of inequality in mortality by sex for population groups defined by area-level deprivation deciles, England, 2006/2008-2014/2016.
Source: Authors' elaborations on data from Office for National Statistics (2018a,b).

Figure S13 shows the results of the Shapley value decomposition of S^P by IMD decile in England.

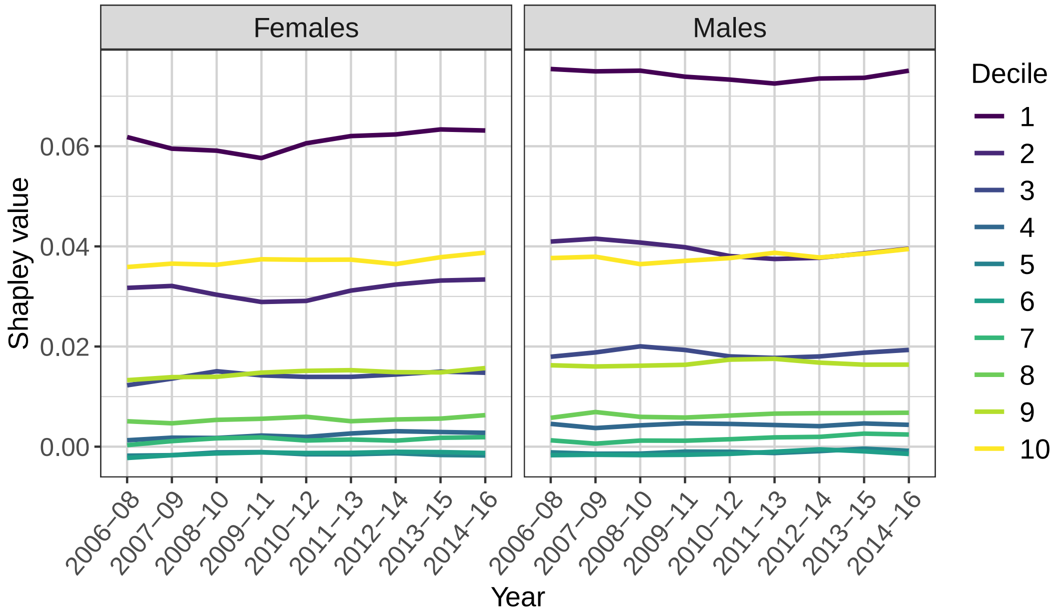

Figure S13: Shapley values of the pairwise non-overlap index (S^P) by IMD deciles for England, 2006-2008 to 2014-2016.
Source: Authors' elaborations on data from Németh et al. (2021) and Human Mortality Database (2023).

Figure S14 shows the results of the decomposition of the changes in the S^P between 2006-2008 and 2014-2016 for England. The decomposition distinguishes between two separate components: mortality changes versus changes in the composition of the population. The first can be further decompose into the changes of the mortality of each group.

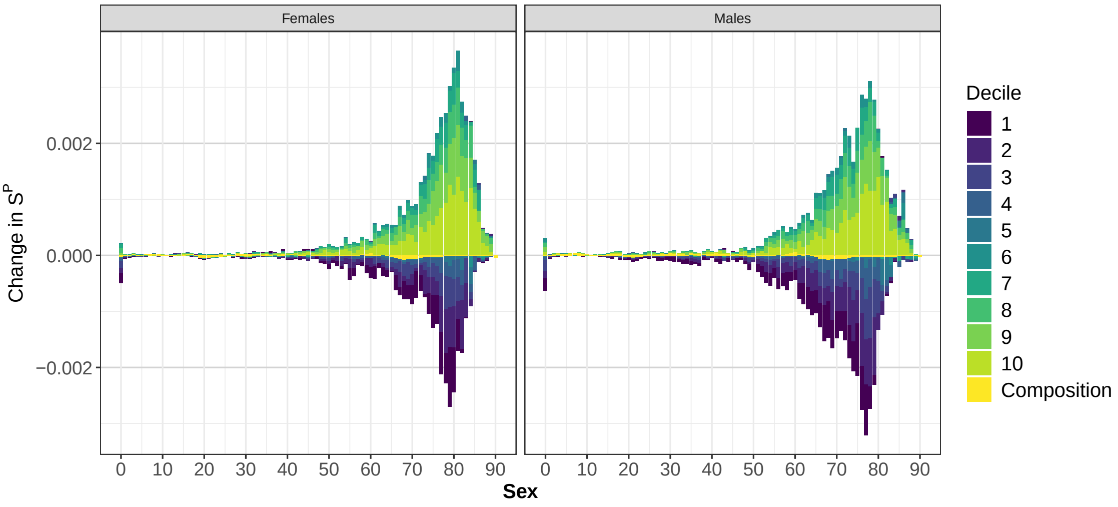

Figure S14: Decomposition by age and IMD decile of the changes in the S^P between 2006-2008 and 2014-2016 for England.
Source: Authors' elaborations on data from Office for National Statistics (2018a,b).

Figure S15 shows the results of the decomposition of the changes in the RII between 2006-2008 and 2014-2016 for England. Same as for the S^P, the decomposition distinguishes between two separate components: mortality changes versus changes in the composition of the population.

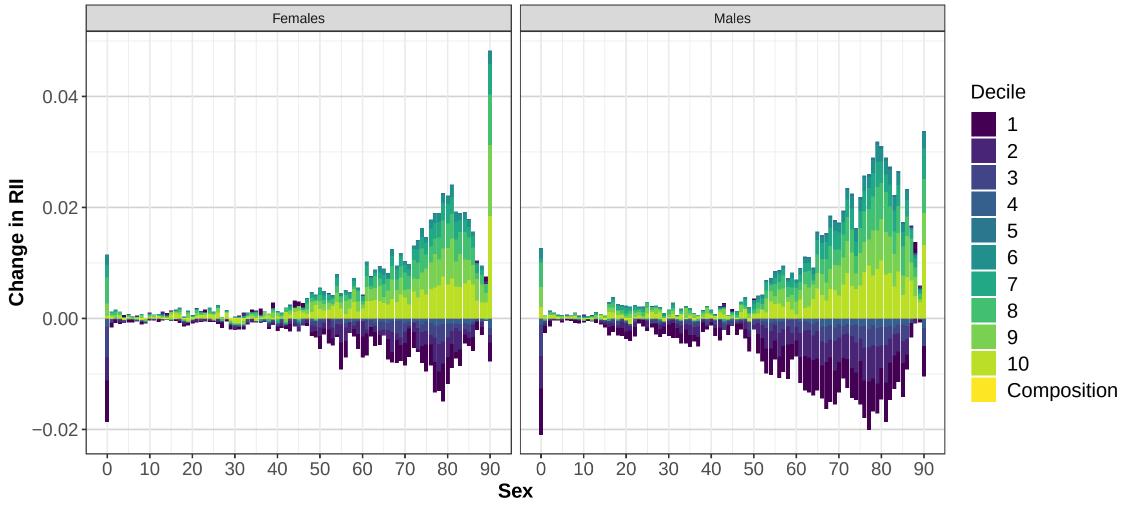

Figure S15: Decomposition by age and IMD decile of the changes in the RII of the age-standardized mortality rates between 2006-2008 and 2014-2016 for England.
Source: Authors' elaborations on data from Office for National Statistics (2018a,b).

Figure S11 shows the trends in four multi-group measures of distributional dissimilarity: pairwise non-overlap index (S^P), total non-overlap index (S^T), stratification index (SI) and pairwise out-survival probability index (OV) for the example by IMD decile presented in the main text.

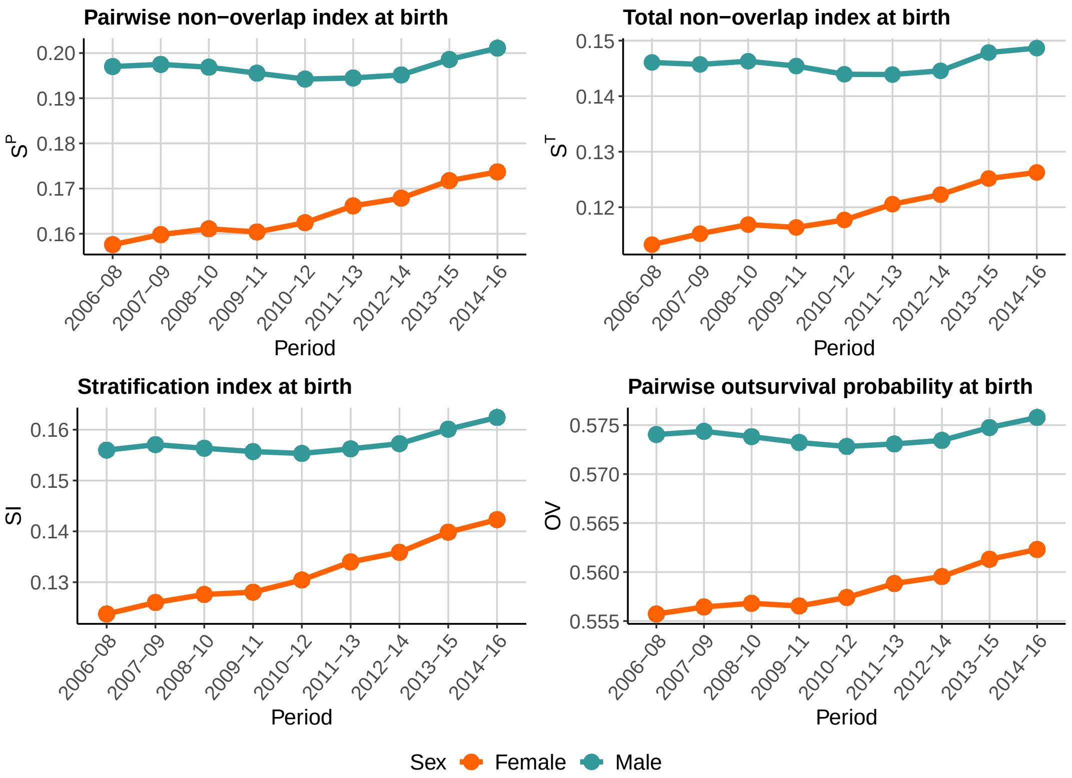

Figure S16: Trends in measures of multi-group distributional dissimilarity in mortality by sex for population groups defined by area-level deprivation deciles, England, 2006-2008 to 2014-2016.
Source: Authors' elaborations on data from Office for National Statistics (2018a,b).

Stepwise decomposition

Formally, let $\boldsymbol{m}_t^i$ and $\boldsymbol{w}_t^i$ denote two vectors of length m containing the age-specific mortality rates and population weights of group $i = 1, 2, .., n$ at time t, respectively. Also, let $\boldsymbol{\theta}_t = [\boldsymbol{m}_t^1, \boldsymbol{m}_t^2, ..., \boldsymbol{m}_t^n, \boldsymbol{w}_t^1, \boldsymbol{w}_t^2, ..., \boldsymbol{w}_t^n]$ denote the population vector of length $2nm$ at time t, and let $f(\boldsymbol{\theta}_t)$ denote the summary measure of interest (S^T or RII). We wish to decompose the between-population difference between time t_1 and t_2, $\Delta_{12} = f(\boldsymbol{\theta}_1) - f(\boldsymbol{\theta}_2)$, into mortality (M-effect) and population (P-effect) contributions, that is $\Delta_{12} = \Delta_{12}^M + \Delta_{12}^P$. The stepwise decomposition method proceeds by replacing the elements of $\boldsymbol{\theta}_2$ with elements of $\boldsymbol{\theta}_1$ one at a time. Following the notation of Jdanov et al. (2017), let $\tilde{\boldsymbol{\theta}}_{12}^j = \left[m_{1,1}^1, \ldots, m_{j,1}^1, m_{j+1,2}^1, \ldots, m_{m,2}^1, \boldsymbol{m}_2^2, ..., \boldsymbol{m}_2^n, \boldsymbol{w}_2^1, \boldsymbol{w}_2^2, ..., \boldsymbol{w}_2^n\right]$ denote the vector at time t_2 after replacement of the first j elements corresponding to those at time t_1, with $\tilde{\boldsymbol{\theta}}_{12}^0 = \boldsymbol{\theta}_2$. Then:

$$\Delta_{12} = \sum_{j=1}^{nm} \left[f(\tilde{\boldsymbol{\theta}}_{12}^j) - f(\tilde{\boldsymbol{\theta}}_{12}^{j-1})\right] + \sum_{j=nm+1}^{2nm} \left[f(\tilde{\boldsymbol{\theta}}_{12}^j) - f(\tilde{\boldsymbol{\theta}}_{12}^{j-1})\right] = \Delta_{12}^M + \Delta_{12}^P.$$

The estimated M and P effects may differ slightly depending on the direction of the decomposition (i.e. whether elements of the vector $\boldsymbol{\theta}_2$ are replaced by those in $\boldsymbol{\theta}_1$ or viceversa), therefore it is customary to run the decomposition in both directions and average the results. For a graphical representation of the stepwise decomposition method, please refer to van Raalte and Nepomuceno (2020).

References

Feehan, D. M. and Wrigley-Field, E. (2021). How do populations aggregate? *Demographic Research*, 44:363.

Human Mortality Database (2023). Max Planck Institute for Demographic Research (Germany), University of California, Berkeley (USA), and French Institute for Demographic Studies (France). Available at www.mortality.org (data downloaded on 02/10/2023).

Jdanov, D. A., Shkolnikov, V. M., van Raalte, A. A., and Andreev, E. M. (2017). Decomposing current mortality differences into initial differences and differences in trends: The contour decomposition method. *Demography*, 54(4):1579–1602.

Németh, L., Jasilionis, D., Brønnum-Hansen, H., and Jdanov, D. A. (2021). Method for reconstructing mortality by educational groups. *Population Health Metrics*, 19(1):34.

Office for National Statistics (2018a). Life table by single year of age sex and deprivation deciles in England, between 2006 to 2008 and 2014 to 2016.

Office for National Statistics (2018b). Number of deaths and populations in deprivation decile areas by sex and single year of age, England and Wales, registered years 2001 to 2017.

Shi, J., Aburto, J. M., Martikainen, P., Tarkiainen, L., and van Raalte, A. (2023). A distributional approach to measuring lifespan stratification. *Population Studies*, 77(1):15–33. doi: 10.1080/00324728.2022.2057576.

van Raalte, A. A. and Nepomuceno, M. R. (2020). *Decomposing Gaps in Healthy Life Expectancy*, pages 107–122. Springer International Publishing.

Vaupel, J., Bergeron-Boucher, M.-P., and Kashnitsky, I. (2021). Outsurvival as a measure of the inequality of lifespans between two populations. *Demographic Research*, 44:853–864.

Zhou, X. (2012). A nonparametric index of stratification. *Sociological Methodology*, 42(1):365–389.

Zhou, X. and Wodtke, G. T. (2019). Income stratification among occupational classes in the united states. *Social Forces*, 97(3):945–972.
